# Behavioural Differences in Dogs with Atopic Dermatitis Suggest Stress Could Be a Significant Problem Associated with Chronic Pruritus

**DOI:** 10.3390/ani9100813

**Published:** 2019-10-16

**Authors:** Naomi D. Harvey, Peter J. Craigon, Stephen C. Shaw, Sarah C. Blott, Gary C.W. England

**Affiliations:** 1 School of Veterinary Medicine and Science, The University of Nottingham, Leicestershire LE12 5RD, UKNaomi.Harvey@nottingham.ac.uk (G.C.W.E.); 2 UK Vet Derm, 16 Talbot Street, Whitwick, Leicestershire LE67 5AW, UK

**Keywords:** clinical animal behaviour, dermatology, stress, questionnaire

## Abstract

**Simple Summary:**

Canine atopic dermatitis (cAD) is a common allergic skin condition in dogs that causes long-term itching; it is similar to eczema in people. The overall quality of life in dogs with cAD is known to be reduced, and humans with eczema report significant psychological burdens from itching that increase stress levels and can lead to the development of additional mental health problems. We tested whether dogs with cAD would display more problem behaviours (that could be indicative of stress) than would healthy controls. Behavioural data were gathered directly from owners using a validated dog behaviour questionnaire for 343 dogs with a diagnosis of cAD and 552 healthy controls, and scores were also provided for the severity of itching experienced by their dog. The results showed that itch severity in dogs with cAD was associated with increased frequency of behaviours often considered problematic, such as: mounting, chewing, hyperactivity, coprophagia (eating faeces), begging for and stealing food, attention-seeking, excitability, excessive grooming and reduced trainability. Whilst we cannot know whether itching directly caused these differences, the behaviours that were associated with itch severity are of a type that are often considered by behavioural biologists to be indicative of stress. Further investigation is warranted, and stress reduction could be helpful when treating dogs with cAD.

**Abstract:**

Canine atopic dermatitis (cAD) is a common allergic skin condition in dogs that causes chronic pruritus. The overall quality of life in dogs with cAD is known to be reduced, and human patients with pruritic conditions report significant psychological burdens from pruritus-induced stress, and atopic dermatitis is associated with significant psychopathological morbidities. We tested the hypothesis that dogs with cAD would display more problem behaviours that could be indicative of stress than would healthy controls. Behavioural data were gathered directly from owners using a validated dog behaviour questionnaire for 343 dogs with a diagnosis of cAD and 552 healthy controls, and scores were also provided for their dog’s pruritus severity. Regression modelling, controlling for potential confounding variables (age, sex, breed, neuter status or other health problem(s)) showed for the first time that pruritus severity in dogs with cAD was associated with increased frequency of behaviours often considered problematic, such as mounting, chewing, hyperactivity, coprophagia, begging for and stealing food, attention-seeking, excitability, excessive grooming, and reduced trainability. Whilst causality cannot be ascertained from this study, the behaviours that were associated with pruritus severity are redirected, self/environment-directed displacement behaviours, which are often considered indicative of stress. Further investigation is warranted, and stress reduction could be helpful when treating dogs with cAD.

## 1. Introduction

Canine atopic dermatitis (cAD) is a chronic allergic skin condition that causes sustained periods of pruritus (itching), inflammation and secondary infections [1]. Pruritus is an aversive subjective sensation which provokes a response to scratch [2]. Pruritic conditions in humans, including atopic dermatitis (hAD), have been repeatedly shown to negatively impact upon the quality of life of those affected [2,3,4]; and cAD has been documented to negatively impact the quality of life of both dogs and their owners [5,6]. Human patients with pruritic conditions report significantly higher psychological stress compared to non-pruritic controls, which is linearly associated with pruritus severity [7]. Furthermore, atopic dermatitis in humans is associated with significant psychopathological morbidities [8,9,10,11]. As such, it is likely that cAD may also have psychological consequences for dog behaviour in terms of stress or anxiety.

There is growing evidence that chronic stress interacts variously with personality, behaviour, health and the immune system in both humans and non-human primates [12]. Deciding which is the causative agent, and which is the response variable remains debatable, however, and the answer is not likely to be a simple one. Behaviour itself can affect physiological factors, such as immunity, whilst at the same time immunity can affect behaviour [13]. Lopes [13] characterises the potential links between immune traits and behaviour as being of three types: (1) Behavioural traits affecting immune traits, for example greater exploratory behaviour leading to a greater exposure to antigens or greater access to food and consequent different gut microbiota; (2) immune traits affecting behavioural traits resulting in behaviour changes indicative of illness; (3) an external or internal mechanism that affects one can simultaneously affect the other, causing changes in both immune response and behaviour. Stress (environmental and psychological) is a factor that disrupts homeostasis, affecting both immune system changes and behavioural changes, with chronic stress being implicated as a risk factor for numerous conditions from cardiovascular disease to cancer [14]. In humans, chronic stress has also been linked to the development of different psychopathologies, including Alzheimer’s disease, Post-Traumatic Stress Disorder [15], and depression [16].

In addition to impacting upon immune regulation, stress disrupts homeostasis, and whilst acute homeostatic changes as a result of a stressor can be adapted to, chronic disruption leads to distress and disease [17,18]. As Hendrix [19] indicates, since the earliest day of psychoanalysis, skin conditions have been regarded as a sign of psychological ‘distress’. Evidence in support of this supposition now exists, with a growing number of studies showing that the “mind does matter” when it comes to skin conditions [20], including, but not limited to, atopic dermatitis [21,22]. Studies with murine models have shown abnormal skin barrier function can be induced by emotional stress, and can be reversed by administration of anxiolytics [23,24]. The link between psychological distress and skin barrier function has also been evidenced in humans, supporting the pathophysiological paradigm of stress as an inducer of impaired epidermal function, which could precipitate or worsen inflammatory dermatoses [20].

Potentially complicating matters are individual differences in stress response and ability to cope with stress. Factors such as an individual’s personality type (where personality is defined as consistent inter-individual differences in behaviour) may impact their vulnerability to stress-induced pathologies. Atopic allergies, such as asthma and allergic dermatitis in humans, have been associated with increased scores for emotional sensitivity [25], lower impulsiveness and greater aggression in relation to irritation [26]. In dogs specifically, it has been found that dogs that were considered to be “well behaved” by their owners lived longer but no behavioural traits were linked to specific immune conditions [27]. However, Non-social fear and Separation anxiety as scored by owners on the canine behavior and research questionnaire (C-BARQ) predicted both the incidence and severity of skin problems in adult dogs [27], although the exact nature of these ‘skin problems’ was not specified.

In contrast to the studies showing causative links between stress and disease status, it has also been suggested that individuals experiencing physical vulnerability, such as injury or disease, may be more likely to pessimistically interpret their environment, which with chronic vulnerability could lead to the development of anxiety disorders [28]. When considering the link between stress and disease status, it is highly plausible to conclude that the stress experienced from one disease or injury state could lead to secondary comorbidities. Indeed, chronic systemic health conditions, such as chronic pain, cardiovascular disease, and cancer, have been associated with the secondary occurrence of mood disorders, such as depression (*for a comprehensive review see* Reference [29]).

The aim of this study was to ascertain whether, and how, dogs with a diagnosis of cAD (cases) would differ behaviourally from dogs of the same breeds with no skin health problems (controls). Whilst causation cannot be established retrospectively, we tested two competing hypotheses based on what we would expect to see if psychological stress was primarily or secondarily associated with the dogs’ skin allergies: (1) If psychological stress was primarily associated (causative) with skin allergies we hypothesised that dogs with skin allergies would have higher trait-level scores for neurotic/fearful scales, characterised by higher scores on fear-based traits potentially reducing their ability to cope with stressors; and (2) if psychological stress develops secondarily as a result of pruritus, we hypothesised that dogs with more severe pruritus from skin allergies would exhibit more stress-related behavioural problems and lower scores for trainability (a score that requires a dog to attend to its owner and sustain its attention to training tasks) and would not differ on trait-level scores for neurotic/fearful scales.

## 2. Materials and Methods

### 2.1. Subjects

All of the dogs in the study were recruited from the pet-owning population as part of the Itchy Dog Project (www.itchydogproject.co.uk). The project was advertised in relevant media (i.e., Vet Record, the Vet Times and dog magazines), social media (Facebook and Twitter), and was listed on The Dog Science Group webpage and The Kennel Club BARC site. Additionally, The Kennel Club sent direct emails out to registered owners of Golden and Labrador Retrievers containing an invitation to take part. Owners were invited to take part no matter what the condition of their dog’s skin; it was made clear that data was required on both healthy dogs and dogs with skin problems. Participation was limited to purebred Golden and Labrador Retrievers as this was part of a larger project aiming to investigate the genetic and environmental risk factors for cAD in these two breeds. Owner participation was entirely voluntary, and consent was gained from each participant as part of the online registration process. Owners first registered for the Itchy Dog Project and completed a questionnaire regarding their dog’s skin health (the canine atopic dermatitis research questionnaire: cAD-RQ [30]), as part of which they were asked to score their dog on a modified Edinburgh pruritus scale [31] to provide a measure of pruritus severity (modifications were minor wording changes and additional descriptions of itch related behaviour to further define the categories). Once they had completed the cAD-RQ, owners received an email inviting them to complete the canine behaviour and research questionnaire (C-BARQ [32]) and containing relevant instructions and an Itchy Dog Project code linking their answers to the project. Owners were blinded to the study’s hypotheses. The C-BARQ was completed by owners of 343 dogs with a veterinary diagnosis of cAD (106 Golden Retrievers and 237 Labradors), and 552 controls (188 Golden Retrievers and 364 Labradors); 895 dogs in total. The sample comprised 422 males, and 473 females, and the mean age for cases was 6.35 years (±SD 2.78), whilst the mean age for controls was 5.92 years (±SD 2.32). A total of 813 dogs (91%) were kennel club registered, and 820 (92%) were from the United Kingdom, 47 (5%) were from the United States, with 28 (3%) from other countries.

Cases had lived with their condition for an average of 4.59 years (SD ± 2.75; age at the time of questionnaire completion minus age when clinical signs first started). Owners were also asked if their dog had had any other (other than itchy skin/ear conditions) health problems or injuries in the past three months; this was a yes/no response, and was tested as an additional predictor in all models compared to the control, for whether it had an impact on any behaviour scores. Amongst Labradors only, chocolate coat colour differed in distribution between cases and controls, with 14.6% of Labrador cases being chocolate coloured (n = 50) compared to 9.2% of controls being chocolate coloured (n = 51).

### 2.2. cAD Phenotype Characterisation

Cases and controls were identified based upon their answers to the cAD-RQ [30]. Cases were dogs that owners reported had received a veterinary diagnosis of cAD. Controls were ≥3 years of age, had never received a diagnosis of canine atopic dermatitis or skin allergies, had never had red, patchy, hairless, rough, swollen or discoloured skin, and had never exhibited signs of abnormal itchiness (frequent and recurrent rubbing, licking, chewing or scratching of the same areas).

### 2.3. The C-BARQ

The C-BARQ constitutes 100 questions scored on either a 5-point ordinal scale, which are scored according to either intensity or frequency of the behaviour described. A score of 0 indicates a total absence of the behaviour described; whilst a 4 indicates the behaviour is seen at the most extreme frequency or intensity. Seventy-eight of the items can be reliably combined to form 14 scale scores, calculated by averaging the items within them. These 14 scale scores are described according to the items which comprise them as follows as per McGreevy et al. [33]:

- *Stranger-directed aggression (severity scale)*: Dog shows threatening or aggressive responses to strangers approaching or invading the dog’s or the owner’s personal space, territory, or home range.
- *Owner-directed aggression (severity scale)*: Dog shows threatening or aggressive responses to the owner or other members of the household when challenged, manhandled, stared at, stepped over, or when approached while in possession of food or objects.
- *Dog-directed aggression (severity scale)*: Dog shows threatening or aggressive responses when approached directly by unfamiliar dogs.
- *Family dog aggression (severity scale)*: Dog shows aggressive or threatening responses to other familiar dogs in the same household.
- *Stranger-directed fear (severity scale)*: Dog shows fearful or wary responses when approached directly by strangers.
- *Non-social fear (severity scale)*: Dog shows fearful or wary responses to sudden or loud noises, traffic, and unfamiliar objects and situations.
- *Dog-directed fear (severity scale)*: Dog shows fearful or wary responses when approached directly by unfamiliar dogs.
- *Touch sensitivity (severity scale)*: Dog shows fearful or wary responses to potentially painful or uncomfortable procedures, including bathing, grooming, nail-clipping, and veterinary examinations.
- *Separation-related behaviour (frequency scale)*: Dog vocalises and/or is destructive when separated from the owner, often accompanied or preceded by behavioural and autonomic signs of anxiety, including restlessness, loss of appetite, trembling, and excessive salivation.
- *Attachment/attention-seeking (frequency scale)*: Dog maintains close proximity to the owner or other members of the household, solicits affection or attention, and displays agitation when the owner gives attention to third parties.
- *Trainability (frequency scale)*: Dog shows a willingness to attend to the owner and obeys simple commands. Dog is not easily distracted, tends to be a fast learner, responds positively to correction, and will fetch or retrieve objects.
- *Chasing (frequency scale)*: Dog chases cats, birds, and/or other small animals, given the opportunity.
- *Excitability (severity scale)*: Dog displays strong reaction to potentially exciting or arousing events, such as going for walks or car trips, doorbells, the arrival of visitors, and the owner arriving home; has difficulty calming down after such events.
- *Energy level (frequency scale)*: Dog is energetic, “always on the go”, and/or playful.

In addition to the 14 scale scores, there are 22 miscellaneous items, which are all scored on a frequency scale (Table 1).

### 2.4. Statistical Analysis

Each population-level C-BARQ score was visually inspected, and frequency analysed before beginning full statistical analysis. Where C-BARQ scores were zero-inflated with less than 5% of the population scoring ≥1 that score was not evaluated further, whilst remaining zero-inflated scores that showed minimal variance between 1–4 were turned into binary variables around their median.

Multivariable regression models were used to analyse the data with each individual C-BARQ score acting as the dependent variable in its own model. Case/Control was included as the main fixed effect to evaluate any possible relationship between cAD diagnosis and behaviour, with potential confounding variables (age, sex, breed, neuter status or other health problem(s)) controlled for at the *p* < 0.05 level. Linear, logistic or Poisson models were used depending on whether the C-BARQ score had a near-normal distribution, was binary, or right-skewed, respectively. Models were checked for fit using diagnostics appropriate to the model used, including: Visual checks of ‘normal’ distribution of residuals using P-P plots, homoscedasticity and VIF values for multicollinearity in linear models, Omnibus tests and Pearsons Chi-square goodness of fit for Poisson models, pseudo-R^2^ values and Hosmer-Lemeshow Chi-square for logistic regressions. All models were fitted in SPSS v.21 (IBM Corp. SPSS Inc. Chicago, IL, USA) using forwards stepwise regression methods with variables remaining in the model if significant to *p* < 0.05. To control for multiple testing, *Q*-values were calculated in R using the package *qvalue* [34,35], and a *q*-value cut-off of *Q* < 0.05 was set to determine significance.

## 3. Results

Four miscellaneous scores (urine marking; emotional urination; urination when left alone; defaecation when left alone) and one scale score (stranger-directed aggression) were excluded from the analysis, due to a lack of variation with 95% or more of the population scoring 0. Median scores on the Edinburgh Pruritus Scale were 1 for controls (interquartile range 1 to 1, minimum 1, maximum 4) and 4 for cases (interquartile range 3 to 4, minimum 1, maximum 6).

The results of the analyses on the remaining C-BARQ scores (Table 2, Table 3 and Table 4) revealed a number of significant differences between cases and controls, that remained significant after correction for multiple testing (*Q* < 0.05). Each of these associations was replicated, and in many cases strengthened, when the Edinburgh Pruritus Scale (EPS) was used as the main effect in the place of case/control status. Using a *Q*-value threshold of *Q* < 0.05, 10 of the 12 variables with case/control as the main effect were retained as statistically significant, and 14 out of 15 with EPS as the main effect were retained.

**Predictions based on Hypothesis** **1.** *Suggested that dogs with cAD would have more generally ‘anxious’, neurotic personality types, with higher scores than controls for scales relating directly to fear and anxiety. However, we found no statistical difference for scales reflecting fearfulness or environmental anxiety, such as stranger-directed fear, dog-directed fear, separation-related problems or non-social fear, and no differences were found in any aggression-related traits (Table 4)*.

**Predictions based on Hypothesis** **2.** *Theorised that pruritic dogs would be more likely to display problem behaviour and would score lower on trainability (a trait for which focus is required), due to the stress-inducing nature of chronic pruritus as documented in studies of people with atopic dermatitis [25]. The results from comparisons of C-BARQ scores to the Edinburgh Pruritus Scale support this hypothesis, with higher scores of itch severity being associated with higher C-BARQ scores for chewing objects, stealing food, excitability, touch sensitivity, mounting, pulling on the leash, coprophagia, hyperactivity/restlessness, begging, and lower scores for trainability (‘escaping’ did not remain significant following Q-value correction). Additional findings were that itch severity was positively associated with the C-BARQ scores for attachment/attention-seeking, other repetitive behaviour, allo-grooming and self-grooming*.

## 4. Discussion

The results of this study support acceptance of Hypothesis 2, that predicted dogs with skin allergies would exhibit more problem behaviours, lower scores for trainability and no differences for generic fearful or environmentally anxious behaviour. The lack of evidence for Hypothesis 1 supports previous findings from Klink and colleagues [36] who found no association between pruritus and generally anxious/fearful behaviour or aggression in pruritic dogs. Here, atopic dogs scored higher than controls for a number of everyday problem behaviours (mounting, coprophagia, hyperactive/restlessness, pulling excessively on the lead), comfort-seeking behaviour (attachment/attention-seeking, begging for food), behaviour likely to be directly related to pruritus (self-grooming, allo-grooming, touch sensitivity), and other repetitive behaviour. Atopic dogs also scored lower for trainability, potentially due to reduced focus from pruritus interrupting/distracting them. Rather than generally having anxious/neurotic personality types as per Hypothesis 1, our results could indicate that the dogs diagnosed with cAD may be experiencing low-level chronic stress as a result of pruritus. In support of this supposition, scores on the Edinburgh Pruritus Scale, a measure of itch severity, were associated with the C-BARQ scores in the same manner as case/control status, implying that pruritus could be the underlying cause for the behavioural differences seen between cases and controls. Pruritus severity was associated with lower scores for trainability and was linearly associated with higher scores for chewing unsuitable objects, mounting, coprophagia, hyperactivity/restlessness, pulling excessively on the lead, attachment/attention-seeking, begging for food, self-grooming, allo-grooming, other repetitive behaviour and touch sensitivity.

Self- and environment-directed behaviours can be a consequence of chronic exposure to aversive conditions in animals, and can lead to behaviours, such as excessive grooming (of themselves and others), coprophagia, hyperactivity, overeating and in extreme situations, stereotypic repetitive functionless behaviour [37]. If the stressor cannot be dealt with, or the motivation cannot be met, animals can express redirected behaviours, such as licking others or inanimate objects or destructive behaviour, where actions are directed towards other stimuli instead [37,38]. The results of this study would suggest that excessive licking of people, other animals or inanimate objects as scored on the C-BARQ may be a redirected behaviour, that in this case is directly associated with pruritus (Allo-grooming, Table 4). Here, pruritus severity was linearly associated with increased scores for multiple redirected, self- and environment- directed displacement behaviours that are likely to be indicative of pruritus-induced psychological stress as has been reported in studies of people with atopic dermatitis [7]. Some of the starkest differences found in the current study included that cases were 2.73 times more likely to exhibit ‘other repetitive behaviour’ than controls, 2.14 times more likely to display mounting behaviour, and were significantly more likely to excessively self-groom. Similar to these findings, McGreevy and colleagues [33] found that small dog breeds exhibit an array of stress/anxiety-related behaviour, including higher scores for mounting, touch sensitivity, begging and attachment/attention-seeking, potentially because their short stature exposes them to a greater number of stressors. Importantly, dogs with cAD did not differ from controls for general trait level scores, such as stranger-directed fear, dog-directed fear, separation-related problems or non-social fear, so do not appear to have neurotic/anxious personality types but do exhibit behavioural signs of increased stress.

**Figure 1:**
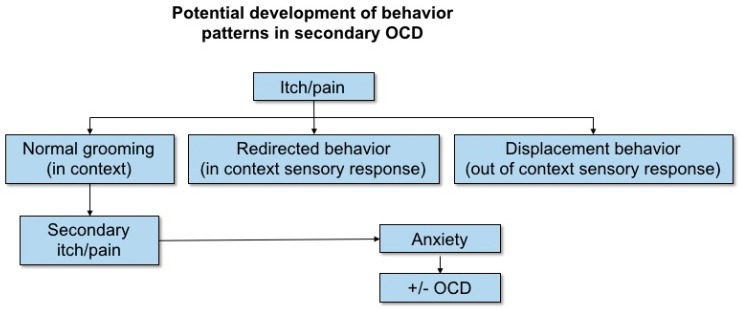
Schematic for how compulsive disorder (OCD) could develop as a secondary condition to dermatological concerns involving pruritus and or/pain. Figure reproduced from Overall, 2013 [39] page number 276, chapter 7, with permission of the rights holder, Elsevier.

The results of this study provide evidence in support of the theory that pruritic dermatoses could lead to the development of secondary compulsive disorders (otherwise known as obsessive-compulsive-disorder, OCD) in dogs [39] (Figure 1). Compulsive disorder in dogs is characterised by a high incidence of over-grooming/self-mutilation, locomotor behaviours, such as spinning/chasing, hallucinatory problems, such as fly catching and shadow-chasing, pica, and to a lesser extent, coprophagia [40]. Whilst the dogs in this study cannot be classified as exhibiting OCD at this stage, those suffering from pruritus did exhibit increased redirected and displacement behaviours, reduced trainability (which may be considered a sign of cognitive impairment, e.g., Reference [41]), and increased comfort-seeking (begging and attachment/attention-seeking). Given these results and what we know about the stress-inducing component of pruritus from human studies [2,3,4,7], it is likely that psychological stress caused by pruritus could have a pathogenic impact on cAD, however, well-controlled studies would be necessary to confirm a causal relationship.

Due to the fact that chronic stress interacts with health in a bi-directional manner [29], and the nature of stress as an inducer of impaired epidermal function [20], it is plausible to conclude that psychological stress-induced by allergic pruritus would further disrupt the skin barrier function, thus, worsening the inflammatory dermatoses. Such a process could mean that the dermatoses becomes self-perpetuating, lengthening the period of a flare even once the initial allergen has been removed. Emotional stress has been implicated in the development and exacerbation of psoriasis in humans, potentially worsening its severity and increasing the time to clearance of a flare [42]. Evidence from murine studies where dermatitis was induced, demonstrate that psychological stress (in this case social isolation) can prolong pruritus in the form of idiopathic dermatitis from self-scratching, long after the inducement has been ceased, whilst the application of topical corticosteroid to the challenged skin prevented the later onset of idiopathic dermatitis [43]. Such findings lend support to the therapeutic use of medications that treat the itch and support the skin barrier as a regular practice even between allergen encounters. Additionally, as suggested in Reference [44], treatment of dermatoses in dogs and other animals should include management of environmental stressors; however, the extent to which this is practiced in veterinary settings is currently unknown. It is also possible that for dogs where environmental stressors and pruritus cannot be controlled, that the use of anxiolytics may result in improved skin barrier function.

Negative affective states are counter-productive for training and learning, with animals that are in negative affective states more likely to judge ambiguity (such as human hand signals or cues) negatively [45]. Further, Eysenck proposed that individuals experiencing anxiety or stress are in a state of divided-attention, with information irrelevant to the task, competing with task-relevant information for processing space [46]. It is, therefore, plausible that the persistent sensation of pruritus in dogs affected with cAD divides the dogs cognitive processing abilities, splitting their attention, in addition perhaps to inducing a negative affective state, which could explain the reduced scores on trainability and increased scores for attachment/attention-seeking and food-seeking as forms of comfort-seeking. However, atopic dermatitis is known to disrupt sleep with increasing pruritic severity [10,47], meaning that the reduction in trainability seen here could be secondary to sleep disturbances inducing cognitive impairment [48]. Alternatively, it is possible that some of the associations seen in this study are the result of side-effects from medication prescribed to treat the dermatoses, as dogs with pruritus are much more likely to be treated with antihistamines and glucocorticoids than dogs without pruritus, which both have potential to affect behaviour [36]. Antihistamines, for example, have a sedative effect and have been shown to induce cognitive impairments in learning and decision-making in humans [49]. Klinck and colleagues found that dogs treated with glucocorticoids were more likely to be reactive to loud noises and thunderstorms [36]; however, we found no difference here between cases and controls or pruritus severity for the C-BARQ scale ‘non-social fear’, which includes noise sensitivity. Dogs with epilepsy have also been shown to score lower than healthy controls for trainability and higher for excitability (also measured by the C-BARQ) [41,50], as was found here with the dogs with cAD, although no differences in the other behavioural factors found here were reported for epileptic dogs. The current study was limited by a lack of information on any medications the dogs were currently being treated with, as no data was collected for current medication use, only medication efficacy for cases. However, due to the confounding association of use of glucocorticoids and antihistamines with pruritus [36], only longitudinal studies that evaluate behaviour and pruritus before and after the medication has been used that could potentially impact behaviour, would be able to truly untangle this potential interaction. Given the results presented here, the impact of pruritus and the medications used for treating pruritic conditions on dog behaviour warrants further investigation. Another factor that may have contributed to the reduction in trainability and increase in food-seeking behaviour could be that cases were more likely to be on an ingredient restricted diet. However, we do not know to what extent each dog found their food rewarding, and it is unlikely to explain the full extent of the results here as they were also associated with increasing severity of pruritus, not just case/control status.

In the Labradors, at least part of the behavioural differences seen in cAD cases may be confounded by coat colour. Chocolate Labradors are at greater risk of having cAD [51], and other pruritic skin problems [52], and have also been shown to score higher than black or yellow Labradors on traits, such as excitability and lower for trainability [53]. Any differences in Labradors, due to coat colour should, at least in part, have been controlled for by including the variable ‘breed’ in our models here. This would, however, be an interesting area for future study; at least some of the behavioural differences seen in chocolate Labradors may be associated with their greater incidence of pruritic skin conditions.

Not only are the results of this study important for the welfare and treatment of dogs affected by cAD, but the increased frequency of ‘problem’ or ‘nuisance’ behaviours reported by owners of pruritic dogs could contribute to a break-down in the dog-owner relationship. Problem behaviour and behavioural characteristics of dogs have been shown to be associated with the quality of the dog-owner relationship [54,55], and furthermore, behavioural problems are the main reason given for relinquishing animals to shelters [56]. Combined with the increased financial burden of treating and managing this chronic condition, and reduced quality of life reported by owners of dogs with cAD [6], the problem behaviours are shown by pruritic dogs in this study could disproportionately increase their risk of being relinquished by their owners.

## 5. Conclusions

This study provides evidence of a potential link between the severity of pruritus and behaviour indicative of psychological stress, in dogs with canine atopic dermatitis. The results identified here show that dogs with cAD display more displacement and redirected behaviour, more comfort-seeking and grooming related behaviour and are less trainable than healthy controls, all of which are directly associated with pruritus severity. Dogs with cAD did not differ from controls for trait-level scales associated with fearfulness/neuroticism, and as such Hypothesis 2 was accepted; that chronic stress may be secondarily associated with chronic pruritus. Since causality cannot be established from association studies, well-controlled prospective studies are required to elucidate whether these associations are, in fact, secondarily associated with pruritus. However, given the large body of evidence demonstrating the impact of stress on skin barrier function, and the increased stress reported by human patients with AD it is plausible that psychological stress experienced by dogs with cAD could prolong and exacerbate allergic flares, potentially compounding the disease with idiopathic dermatoses. As suggested by others, these results support the call for treatment of dermatoses in dogs and other animals to include management of environmental stressors to reduce the overall stress burden. Given the potential importance of stress in cAD pathogenesis, and the implications of the behavioural problems demonstrated here for dog welfare and the human-animal bond, further study into the potential stress-inducing nature of pruritus from cAD is warranted.

## Figures and Tables

**Table 1:** The 22 miscellaneous items from the canine behaviour and research questionnaire (C-BARQ) (scored as frequency scales).

CBARQ Score	Wording
Escapes	Escapes or would escape from home or garden given the chance.
Rolls in faeces	Rolls in animal droppings or other ‘smelly’ substances.
Coprophagia	Eats own or other animals’ droppings or faeces.
Chews	Chews inappropriate objects.
Mounts	Mounts objects, furniture, or people.
Begs for food	Begs persistently for food when people are eating.
Steals food	Steals food
Nervous on stairs	Nervous or frightened on stairs.
Pulls on leash	Pulls excessively hard when on the leash.
Urine marks	Urinates against objects/furnishings in your home.
Emotional urination	Urinates when approached, petted, handled or picked up.
Urination when left alone	Urinates when left alone at night, or during the daytime.
Defaecation when left alone	Defaecates when left alone at night, or during the daytime.
Hyperactive/restless	Hyperactive, restless, has trouble settling down.
Compulsive staring	Stares intently at nothing visible.
Snaps at flies	Snaps at (invisible) flies.
Tail chasing	Chases own tail/hind end.
Shadow chasing	Chases/follows shadows, light spots, etc.
Barks persistently	Barks persistently when alarmed or excited.
Self-grooming	Licks him/herself excessively.
Allo-grooming	Licks people or objects excessively.
Other bizarre or repetitive behaviour	Displays other bizarre, strange, or repetitive behaviour(s) (Please describe)

**Table 2:** Results of linear regression models comparing behaviour scores for Labrador and Golden Retrievers with a veterinary diagnosis of canine atopic dermatitis (cases) to Labrador and Golden Retrievers with no skin problems (controls), and to scores on the Edinburgh Pruritus Scale (EPS). Items underlined are considered statistically significant to a false discovery rate (*Q*-value) of less than 5% (<0.05). Predictors remaining in the model were statistically significant to *p* < 0.05. B, the coefficient for the main effect; 95% CI, 95% confidence interval for B; t, the t-statistic (B divided by its standard error); P, *p*-value for B.

C-BARQ Score	Predictors	Main Effect: Diagnosis (Case vs. Control)				Main Effect: Itch Severity (EPS)			
		B	95% CI	t	P	B	95% CI	t	P
Trainability	Breed, Sex	−0.10	−0.18 to −0.03	−2.92	0.004	−0.04	−0.07 to −0.02	−3.51	<0.001
Excitability	None	0.09	−0.20 to 0.19	1.59	0.112	0.05	0.01 to 0.09	2.65	0.008
Attachment/attention-seeking	Age	0.19	0.09 to 0.30	3.70	<0.001	0.08	0.05 to 0.12	4.77	<0.001
Chasing	Breed, Age	0.09	−0.05 to 0.22	1.25	0.211	0.04	−0.00 to 0.09	1.82	0.069
Energy	Age	0.07	−0.05 to 0.20	1.14	0.254	0.03	−0.01 to 0.07	1.31	0.191

**Table 3:** Right skewed integer data analysed with a Poisson regression model. Statistics describe models with diagnosis (case) and scores on the Edinburgh Pruritus Scale (EPS) as the main effect. Items underlined are considered statistically significant to a false discovery rate (*Q*-value) of less than 5% (<0.05). Predictors remaining in the models were statistically significant to *p* < 0.05. B, the coefficient for the main effect; 95% CI, 95% confidence interval for B; Wald, chi-square value associated with B; P, *p*-value for B.

C-BARQ Score	Predictors	Main Effect: Diagnosis (Case vs. Control)				Main Effect: Itch Severity (EPS)			
		B	95% CI	Wald	P	B	95% CI	Wald	P
Rolling	Breed, Sex, Neutered	0.04	−0.07 to 0.15	0.48	0.488	0.02	−0.02 to 060	1.26	0.263
Coprophagia	Sex, Neutered	0.15	0.03 to 0.27	5.79	0.016	0.64	0.03 to 0.10	10.01	0.001
Begging	Breed, Neutered	0.25	0.14 to 0.36	20.49	<0.001	0.09	0.06 to 0.13	26.73	<0.001
Pulls leash	Sex, Age	0.25	0.13 to 0.37	17.36	<0.001	0.08	0.04 to 0.12	16.49	<0.001
Self-grooms	None	1.89	1.69 to 2.01	348.32	<0.001	0.49	0.44 to 0.54	422.98	<0.001

**Table 4:** Zero-inflated data, converted to binary (0, >0) analysed via logistic regression. Statistics describe models with diagnosis (case) and scores on the Edinburgh Pruritus Scale (EPS) as the main effect. Underlined items are considered statistically significant to a false discovery rate (*Q*-value) of less than 5% (<0.05). Predictors remaining in the model were statistically significant to *p* < 0.05. B, the coefficient for the main effect; 95% CI, 95% confidence interval for B; Wald, chi-square value associated with B; P, *p*-value for B. Behaviour scores in this table have been grouped by type for ease of interpretation.

Type	Scores	Predictors	Main Effect: Diagnosis (Case)				Main Effect: Itch Scale			
			OR	95% CI	Wald	P	OR	95% CI	Wald	P
*Fear/anxiety/aggression traits*										
	Non-Social Fear	Breed	1.04	0.79 to 1.37	0.08	0.785	1.03	0.93 to 1.13	0.28	0.599
	Owner Dir. Agg.		1.20	0.81 to 1.78	0.79	0.374	1.10	0.97 to 1.25	2.05	0.152
	Dog Dir. Fear		1.27	0.97 to 1.66	2.93	0.087	1.06	0.97 to 1.17	1.75	0.186
	Dog Dir. Agg.	Age, Sex, Sex * Neutered	1.14	0.86 to 1.51	0.81	0.368	1.05	0.95 to 1.16	0.98	0.323
	Stranger Dir. Fear	Age, Breed	0.96	0.71 to 1.31	0.06	0.814	0.97	0.87 to 1.08	0.27	0.605
	Family Dog Agg.		0.79	0.56 to 1.11	1.85	0.174	0.99	0.89 to 1.12	0.00	0.953
	Sep. Rel. Beh.		0.95	0.73 to 1.25	0.12	0.735	1.01	0.92 to 1.11	0.05	0.826
	Stair fear		1.10	0.76 to 1.59	0.24	0.621	1.09	0.96 to 1.23	1.72	0.189
*Redirected/displacement behaviour*										
	Chewing	Age	1.40	1.06 to 1.85	5.64	0.018	1.14	1.04 to 1.25	7.53	0.006
	Mounting	Breed, Sex	2.14	1.28 to 3.55	8.55 *	0.003	1.14	1.02 to 1.28	5.23	0.022
	Hyper/restless	Age	1.64	1.18 to 2.28	8.85	0.003	1.20	1.08 to 1.34	11.43	0.001
	Steals food	Breed, Neutered	1.18	0.89 to 1.57	1.39	0.238	1.14	1.04 to 1.25	7.35	0.007
	Allo-grooming	Age, Breed	1.88	1.41 to 2.52	18.14	<0.001	1.27	1.15 to 1.40	22.68	<0.001
*Abnormal/repetitive behaviour*										
	Staring	Neutered	1.26	0.92 to 1.72	2.04	0.153	1.09	0.99 to 1.21	2.82	0.093
	Snaps at flies		0.79	0.54 to 1.17	1.38	0.240	0.94	0.83 to 1.08	0.71	0.401
	Chases tail	Age, Breed	0.97	0.72 to 1.31	0.05	0.830	1.05	0.95 to 1.16	0.81	0.369
	Chases shadows	Age, Breed	1.27	0.90 to 1.79	1.82	0.178	1.09	0.97 to 1.23	2.15	0.142
	Other Rep. Beh.		2.73	1.79 to 4.15	21.91	<0.001	1.44	1.27 to 1.64	10.81	<0.001
*Other/problem behaviour*										
	Touch Sens.		1.38	1.05 to 1.82	5.39	0.020	1.12	1.02 to 1.23	6.06	0.014
	Barking	Breed	1.24	0.94 to 1.63	2.37	0.124	1.09	0.99 to 1.20	3.76	0.052
	Escapes		1.21	0.92 to 1.59	1.87	0.171	1.11	1.01 to 1.22	4.90	0.027

* indicates main effect statistic from an interaction between diagnosis and neuter status. Behaviours are categorised by type given in italics.
